# Characteristics of Pediatric Dental Injuries and Predictive Factors for Long Treatment Duration

**DOI:** 10.1002/cre2.70218

**Published:** 2025-09-08

**Authors:** Yuki Sakamoto, Mineko Baba, Shinya Kosinuma, Kazuki Takaoka, Mami Nakamura, Masahito Hitosugi

**Affiliations:** ^1^ Department of Legal Medicine Shiga University of Medical Science Otsu Shiga Japan; ^2^ Center for Integrated Medical Research Keio University School of Medicine Shinjuku Tokyo Japan; ^3^ Department of Oral and Maxillofacial Surgery Shiga University of Medical Science Otsu Shiga Japan

**Keywords:** child, duration of therapy, maxillofacial injuries, tooth injuries

## Abstract

**Objectives:**

This study was performed to identify the characteristics of oral and maxillofacial injuries in children and determine factors influencing a treatment duration of more than 1 month using medical records.

**Material and Methods:**

This retrospective observational study reviewed medical records of 258 children (< 16 years of age) with maxillofacial trauma treated at a university hospital between 2011 and 2021. Patients with and without tooth fractures or dislocations were compared, and the prevalence of injuries was analyzed across three age groups (0–2, 3–5, ≥ 6 years). Long (> 1 month) and short (< 1 month) treatment durations were compared, and independent predictive factors for prolonged treatment were identified.

**Results:**

Tooth injuries were observed in 204 (79.1%) patients. Deciduous central incisors in the primary dentition and central incisors in the permanent dentition were the most commonly affected. Soft tissue injuries occurred in 165 (64.0%) patients, while maxillofacial fractures were present in 7 (2.7%). Tooth fractures significantly increased with age (*p* < 0.001), while dislocations showed no age‐related differences. Logistic regression revealed that maxillofacial fractures, tooth fractures, and two or more dislocated teeth were independent predictors of prolonged treatment, with odds ratios of 14.666 (1.625–132.359), 2.411 (1.099–5.287), and 2.350 (1.208–4.570), respectively.

**Conclusions:**

Maxillofacial fractures, multiple tooth dislocations, and tooth fractures were confirmed as key factors influencing longer treatment durations. These findings may assist in the early management of maxillofacial injuries and improve communication with pediatric patients and their families.

## 1 Introduction

Oral and maxillofacial trauma is a common public health issue that significantly affects masticatory function and quality of life. Owing to anatomical, physiological, and psychological differences between adults and children, the consequences and management of trauma vary. Children are particularly prone to oral and maxillofacial trauma because of their higher cranial mass‐to‐body weight ratio. A prospective study conducted in Denmark showed that approximately half of all children experience at least one dental injury before finishing school (Andreasen and Ravn 1972). Additionally, a retrospective study from Sweden revealed that 35% of 16‐year‐olds had sustained at least one dental injury (Borssén and Holm 1997). Variations in the head–body relationship and the developmental status of maxillofacial structures, including teeth, contribute to age‐related differences in injury types. Furthermore, oral and maxillofacial trauma in children affects not only the child but also the emotional and financial well‐being of the parents (Feldens et al. 2014).

Dental and dentoalveolar injuries are often overlooked in pediatric oral and maxillofacial trauma because of the challenges posed by clinical and radiological examinations. The presence of unerupted or mixed dentition in children makes it difficult to predict maxillofacial fractures (Gassner et al. 2004). In cases of multiple injuries, oral and maxillofacial management is often delayed because treatment of vital organs is prioritized. Delays in referring patients to oral and maxillofacial surgeons or inadequate management may lead to long‐term complications, such as external inflammatory root resorption, replacement resorption, tooth discoloration, periradicular pathology, and tooth loss (Traebert et al. 2018). Therefore, it is essential for dental practitioners to fully understand the characteristics of maxillofacial trauma in children.

Although several studies have reported on the characteristics and backgrounds of pediatric oral and maxillofacial trauma, few have compared detailed information across specific age groups. One multicenter prospective study collected data from hospitalized patients aged 0 to 18 years with oral and maxillofacial injuries, examining the cause of injuries and the patterns of maxillofacial fractures across different age groups (Segura‐Palleres et al. 2022). The study showed that falls were the leading cause of injury in preschool‐aged children, with the mandibular condyle being the most commonly affected fracture site in preschool and school‐aged groups, and the nose most commonly affected in adolescents (Segura‐Palleres et al. 2022). Another cross‐sectional study in Brazil involving 3489 children aged 3–59 months showed that dental trauma was most frequent in children aged 2–4 years, with crown fractures being the most common injury (Ferreira et al. 2009). A retrospective analysis of a dental trauma emergency clinic indicated that dental trauma most frequently occurred in children aged 6–15 years, while intrusive luxation cases were most commonly diagnosed in children aged 1–5 years (De França Caldas and Burgos 2001). However, no studies have specifically compared the prevalence of oral and maxillofacial injuries across different stages of childhood development.

The management of maxillofacial injuries should be tailored to the age of the patient. Open reduction internal fixation can damage developing teeth and impair osteogenic potential by stripping the periosteum, which may result in scarring and growth restrictions after the placement of metallic plates (Saikrishna and Gupta 2010; Kambalimath et al. 2013). Therefore, most pediatric maxillofacial injuries are managed conservatively, with surgical intervention reserved for more severe cases such as displaced fractures, occlusion interference, orbital involvement, and panfacial fractures, particularly in older children (Patidar et al. 2024). Although conservative treatment is common, maxillofacial injuries can still have significant psychological impacts on both children and their families, negatively affecting oral health‐related quality of life (Tewari et al. 2023). Long treatment durations can exacerbate anxiety and emotional concerns. Identifying predictive factors for extended treatment durations may allow for early interventions to mitigate these psychological effects. While several studies have examined factors influencing prolonged hospital stays or treatment durations in patients with oral and maxillofacial injuries (Hong et al. 2013; Haghparast‐Bidgoli et al. 2013; Machida et al. 2023; Hirobe et al. 2021), no research has focused specifically on long treatment durations in pediatric patients.

The objectives of the present study were to identify the characteristics of oral and maxillofacial injuries in children at different stages of development and to determine the factors influencing a treatment duration of more than 1 month based on medical record data.

## 2 Materials and Methods

### 2.1 Materials

This manuscript was prepared in alignment with the STROBE Statement. This study included pediatric patients (age 0–15 years) with oral and maxillofacial injuries. This hospital [identifying information removed for review] is the only university hospital in the area with specialists available to treat oral and maxillofacial trauma around the clock. More than two‐thirds of patients with oral and maxillofacial trauma in the prefecture are treated at this facility. The department manages approximately 18,000 in‐patients annually, of whom approximately 40–50 are trauma patients. During the 11‐year study period, 258 children were treated for trauma. Hospital records from the Department of Oral and Maxillofacial Surgery at a university hospital were reviewed for the period from 2011 to 2021. All trauma patients aged 15 years or younger who were referred to our department (including those from other institutions) were included in the study. Because our facility is not an advanced emergency and critical care center, patients with severe polytrauma or critical head injuries were not admitted. The following variables were extracted from the patients' medical records:

- Basic information: age and sex.
- Oral and maxillofacial trauma details: time of injury (day or night) and location of impact (indoor or outdoor).
- Location of the injured tooth and type of injury (dislocation or fracture).
- Presence or absence of soft tissue injury.
- Presence or absence of maxillofacial fractures.
- Treatment duration.

### 2.2 Methods

After the overall results had been reviewed, the patients were divided into those with and without tooth fractures or dislocations for comparison. Generally, primary tooth eruption is complete by around 2.5 years of age, and permanent teeth begin to emerge at approximately 6 years of age. Therefore, taking tooth development milestones into account, patients were further divided into three age groups: 0–2 years, 3–5 years, and ≥ 6 years. The prevalence of each type of injury was compared among these groups. Additionally, the patients' backgrounds were analyzed to compare those with long treatment durations (1 month or more) and those with shorter treatment durations. Finally, independent predictive variables for prolonged treatment duration were determined.

### 2.3 Statistical Analysis

The chi‐square test was used to compare frequencies between the two groups. Logistic regression analysis was performed using the forced‐entry method to identify factors influencing the dependent variable. All statistical analyses were performed using SPSS version 23 (IBM Corp., Armonk, NY, USA), with a *p*‐value of < 0.05 considered statistically significant.

## 3 Results

### 3.1 General Characteristics

**Figure 1:**
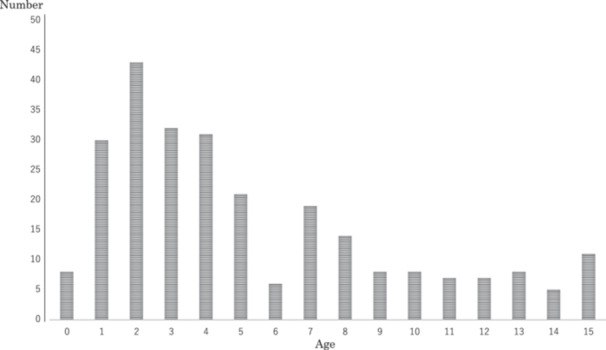
Distribution of all children by age.

In total, 258 patients (150 males, 108 females) aged 0–15 years (median age, 4 years) were included in the study (Figure 1). The median age of the patients was 4 years, with an interquartile range of 2–8 years. The timing of the injuries was as follows: 171 (66.3%) injuries occurred during the daytime, 82 (31.8%) during the nighttime, and 5 (1.9%) at an unknown time. Regarding the location of injury, 137 (53.1%) injuries occurred indoors, 100 (38.8%) outdoors, and 16 (8.1%) in an unknown location. Falls were the most common cause of injury, accounting for 222 (86.0%) cases.

### 3.2 Injury Characteristics

**Figure 2:**
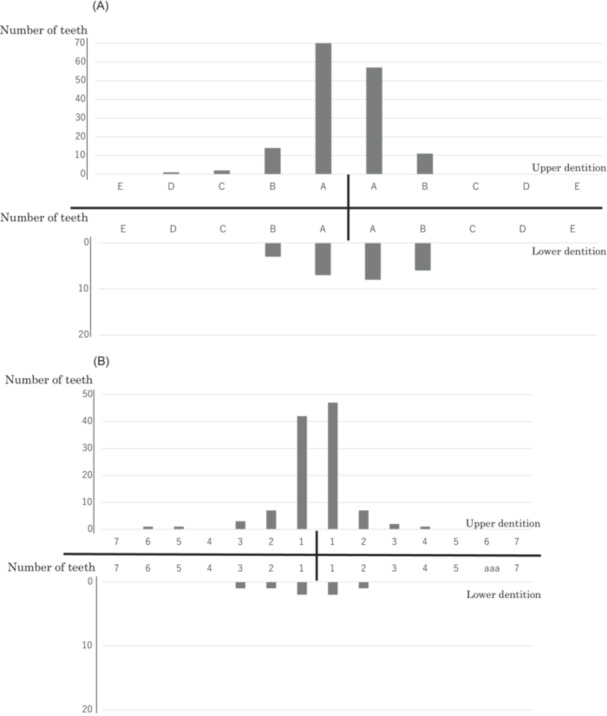
(A) Distribution of injured primary teeth among all children. (B) Distribution of injured permanent teeth among all children.

Tooth injuries were observed in 204 (79.1%) patients. Of these, 150 (58.1%) had tooth dislocations and 46 (17.8%) had tooth fractures. Nine (3.5%) patients presented with both dislocated and fractured teeth, while 71 (27.5%) had two or more dislocated teeth, and another 71 (27.5%) had no tooth damage. The distribution of injured teeth in the primary and permanent dentition is shown in Figure 2A,B. Deciduous central incisors in the primary dentition and central incisors in the permanent dentition were the most commonly affected.

A total of 165 (64.0%) patients had soft tissue injuries, and 7 (2.7%) had maxillofacial fractures.

### 3.3 Comparison of Patients With and Without Tooth Fracture or Dislocation

A comparison of patients with and without tooth dislocations revealed that the prevalence of soft tissue injuries was significantly higher in patients without dislocations (*p* < 0.001) (Table 1). No significant differences were observed in other variables. A similar comparison was made for tooth fractures, which were more common in patients aged ≥ 6 years and less frequent in those aged ≤ 2 years (*p* < 0.001; Table 2). Tooth fractures also occurred significantly more frequently in outdoor settings (*p* = 0.004). No other significant differences were noted.

**Table 1:** Comparison of backgrounds of patients with and without tooth dislocation.

	Teeth discoloration		
	+ (*n* = 150)	− (*n* = 108)	*p*‐value
Age			
0–2	47 (31.3%)	34 (31.5%)	0.964
3–5	48 (32.0%)	36 (33.3%)	
≥ 6	55 (36.7%)	38 (35.2%)	
Sex			
Male	84 (56.0%)	69 (63.9%)	0.203
Female	66 (44.0%)	39 (36.1%)	
Location			
Indoor	71 (47.3%)	66 (61.1%)	0.062
Outdoor	64 (42.7%)	36 (33.3%)	
Unknown	15 (10.0%)	6 (5.6%)	
Time zone			
Day	102 (68.0%)	69 (63.9%)	0.472
Night	45 (30.0%)	37 (34.3%)	
Unknown	3 (2.0%)	2 (1.9%)	
Soft tissue injury			
+	78 (52.0%)	86 (79.6%)	< 0.001
−	72 (48.0%)	22 (20.4%)	

**Table 2:** Comparison of the backgrounds of patients with and without tooth fractures.

	Teeth discoloration		
	+ (*n* = 46)	− (*n* = 212)	*p*‐value
Age			
0–2	2 (4.4%)	79 (37.3%)	< 0.001
3–5	15 (32.6%)	69 (32.5%)	
≥ 6	29 (63.0%)	64 (30.2%)	
Sex			
Male	33 (71.7%)	120 (56.6%)	0.058
Female	13 (28.3%)	92 (43.4%)	
Location			
Indoor	17 (37.0%)	120 (56.6%)	0.004
Outdoor	27 (58.7%)	73 (34.4%)	
Unknown	2 (4.3%)	19 (9.0%)	
Time zone			
Day	32 (69.6%)	139 (65.6%)	0.752
Night	14 (30.4%)	68 (32.1%)	
Unknown	0 (0%)	5 (2.4%)	
Soft tissue injury			
+	24 (52.2%)	140 (66.0%)	0.077
−	22 (47.8%)	72 (34.0%)	

### 3.4 Prevalence of Each Injury by Age Group

The prevalence of injuries was compared across three age groups (Table 3). Although the prevalence of tooth dislocations was similar across all groups, the prevalence of tooth fractures significantly increased with age (*p* < 0.001). Additionally, the prevalence of patients with both tooth dislocation and fracture increased significantly with age (*p* = 0.030), while the prevalence of those with neither injury significantly decreased with age (*p* = 0.002).

**Table 3:** Prevalence of each injury by age group.

	Age group			
	0–2 (*n* = 81)	3–5 (*n* = 84)	≥ 6 (*n* = 93)	*p*‐value
Dislocation	46 (56.8%)	47 (56.0%)	48 (51.6%)	0.964
Tooth fracture	1 (1.2%)	14 (16.7%)	22 (23.7%)	< 0.001
Both tooth dislocation and fracture	1 (1.2%)	1 (1.2%)	7 (7.5%)	0.030
Neither	33 (40.7%)	22 (26.2%)	16 (17.2%)	0.002
Soft tissue injury	52 (64.2%)	50 (59.5%)	63 (67.7%)	0.426

### 3.5 Comparison of Patients With Short and Long Treatment Durations

Patients with short (< 1 month) and long (≥ 1 month) treatment durations were compared based on age and the prevalence of each injury (Table 4). Longer treatment durations were more commonly observed in older children (*p* = 0.08), patients with two or more dislocated teeth (*p* = 0.029), patients with tooth fractures (*p* = 0.007), and patients with maxillofacial fractures (*p* < 0.001).

**Table 4:** Comparison of the prevalence of tooth and maxillofacial injuries between patients with treatment durations of < 1 month or ≥ 1 month.

	< 1 month (*n* = 192)	≥ 1 month (*n* = 66)	*p*‐value
Age			
0–2	69 (35.9%)	12 (18.2%)	0.08
3–5	63 (32.8%)	21 (31.8%)	
≥ 6	60 (31.３%)	33 (50.0%)	
Tooth dislocation (≥ 2)	46 (24.0%)	25 (37.9%)	0.029
Tooth fracture	27 (14.1%)	19 (28.8%)	0.007
Maxillofacial fracture	1 (0.5%)	6 (9.1%)	< 0.001
Soft tissue injury	123 (64.1%)	41 (62.1%)	0.777

To determine the variables associated with longer treatment durations, logistic regression analysis was performed using the following independent variables: age group, presence of two or more dislocated teeth, presence of tooth fractures, presence of maxillofacial fractures, and presence of soft tissue injuries (Table 5). Maxillofacial fractures, tooth fractures, and two or more dislocated teeth were identified as independent predictive factors for longer treatment duration, with odds ratios and 95% confidence intervals of 14.666 (1.625–132.359), 2.411 (1.099–5.287), and 2.350 (1.208–4.570), respectively. This analysis was not affected by multicollinearity; the variance inflation factor ranged from 1.022 to 1.217.

**Table 5:** Results of logistic regression analysis for long treatment duration (≥ 1 month).

	Odds ratio	95% confidence interval	*p*‐value
Age			
0–2	Ref.	Ref.	
3–5	1.461	0.644–3.318	0.364
≥ 6	2.023	0.898–4.559	0.089
Tooth dislocation (≥ 2)	2.350	1.208–4.570	0.012
Tooth fracture	2.411	1.099–5.287	0.028
Maxillofacial fracture	14.666	1.625–132.359	0.017
Soft tissue injury	0.936	0.502–1.744	0.835

## 4 Discussion

In this study, most patients (86.0%) had sustained oral and maxillofacial trauma due to falls. A meta‐analysis indicated that globally, road traffic collisions were the most common cause of maxillofacial trauma among children and adolescents, followed by falls, violence, and sports (Mohammadi et al. 2023). However, a review of 3,385 children under 15 years old showed that falls were the predominant mechanism of injury, accounting for 54.7% of cases (Gassner et al. 2004). Another review of 22,839 children aged 6 years or younger identified falls as the leading cause of traumatic injuries to primary teeth (Patnana et al. 2021). A single‐center retrospective study suggested that in early childhood, falls are the most common cause of maxillofacial trauma (AlAli et al. 2021). Similarly, in infants and preschool children, falls at home were the most frequently reported cause of injury (Kumaraswamy et al. 2009). The risk of oral and maxillofacial trauma due to falls at home or in surrounding areas increases as children learn to walk, play, or eat. Furthermore, the prevalence of falls as a cause of trauma varies by region, with falls being more prevalent in Asian populations (Mohammadi et al. 2023). The prevalence of dental trauma is higher in developing countries than in developed countries (Marcenes et al. 1999), underscoring the need for region‐specific studies on oral and maxillofacial trauma.

Regarding the location of injury, indoor incidents (53.1%) were more common than outdoor incidents (38.8%). A systematic review and meta‐analysis of primary tooth injuries showed that most injuries occurred at home (72%). Children with primary teeth spend the majority of their time at home, raising questions about the adequacy of safety measures in spaces generally considered safe for children. Frequent adult supervision, particularly for younger children, and safe environments are critical to reducing the incidence of oral and maxillofacial injuries.

In this study, soft tissue injuries (63.6%) and tooth dislocations (58.1%) were more prevalent than tooth fractures (17.8%) in children younger than 15 years. The prevalence of maxillofacial fractures was relatively low (2.7%). In general, the prevalence of maxillofacial fractures increases with age (Zimmermann et al. 2005). Among pediatric patients under 16 years of age, the prevalence of maxillofacial fractures ranges from 1% to 14%, and for patients under 5 years old, it ranges from 0.9% to 1.0% (Sawhney and Ahuja 1988). The findings of this study align with these reports. Some studies have suggested that maxillofacial fractures are rare in young children, with incidence rates increasing as children approach adolescence (Segura‐Palleres et al. 2022; Barbosa et al. 2017; Cleveland et al. 2021; Blasco et al. 2020). The lower prevalence of maxillofacial fractures in younger children is likely due to the flexibility of their maxillofacial bones, the lack of ventilation in their paranasal sinuses, and the protective cushioning provided by buccal adipose tissue in infants (Gassner et al. 2004). In this study, the low prevalence of fractures was likely due to the younger age of the patients (median age of 4 years) and the tendency for injuries to result from low‐velocity forces, such as slips and trips, rather than high‐energy trauma such as motor vehicle collisions.

Previous studies have shown that soft tissue injuries are common among pediatric patients with facial trauma, with rates ranging from 54.5% to 70.0% (Patidar et al. 2024; Kotecha et al. 2008). The results of this study are consistent with these findings. Soft tissue injuries often occur in conjunction with facial fractures, with a prevalence of 29%–56%, but they may be overlooked in isolated cases (Haug and Foss 2000). Prompt treatment of soft tissue injuries is essential in children because healing occurs more rapidly than in adults. Correct diagnosis and management of soft tissue injuries should be emphasized.

In this study, the most commonly injured teeth were the incisors. A meta‐analysis of traumatic injuries to primary teeth showed that the maxillary central incisors were the most frequently injured, with a prevalence of 73.9% (Patnana et al. 2021). A cross‐sectional study in Brazil also reported that the upper central incisors were the most affected teeth (Ferreira et al. 2009). An analysis of dental injuries among bicyclists younger and older than 20 years revealed that fractures and dislocations primarily occurred in the incisors for both groups (Tsutsumi et al. 2021). This study confirmed that the frequent occurrence of dental injuries in the incisors is consistent between children and adults.

The results also indicated that the prevalence of tooth fractures increased with age and was rarely observed in children aged 2 years or younger. This increase in the prevalence of tooth fractures is likely due to older children in preschool or school engaging in more activities such as walking, running, and participating in sports, combined with a general decrease in adult supervision. These findings are consistent with the observation that tooth fractures occurred more frequently outdoors than indoors in the present study. As children grow older, the supporting structures around their teeth become less elastic, and greater forces are exerted on the face during activities, resulting in a higher prevalence of tooth fractures.

Tooth dislocation was the second most common injury after soft tissue injury across all age groups, and there were no significant differences in the prevalence of dislocation between age groups. When an impact occurs, younger children's teeth tend to displace apically because their supporting tissues are less resistant to tooth movement. Previous studies have suggested that luxation injuries are more common in primary teeth than in permanent teeth (de Amorim et al. 2011; Assunção et al. 2011). This is due to the elasticity of the supporting structures and the shorter root length of primary teeth (Mahmoodi et al. 2015). Additionally, primary teeth are more closely spaced within a smaller area than are permanent teeth, making an impact more likely to affect multiple teeth in younger children (De França Caldas and Burgos 2001). Furthermore, younger children may lack the motor coordination needed to minimize injuries during falls or impacts.

This study confirmed that having two or more dislocated teeth, tooth fractures, and maxillofacial fractures were significant predictive factors for a longer treatment duration of 1 month or more. Maxillofacial fractures were associated with a particularly high odds ratio (OR). This finding is consistent with previous research, which identified the use of intermaxillary fixation (OR 16.09) and the number of injured teeth (OR 1.52) as independent predictors of longer treatment durations in patients with maxillofacial injuries (Machida et al. 2023). Generally, age, sex, location and nature of the injuries, and other sociodemographic characteristics have been considered factors related to longer hospital stays (Hong et al. 2013; Haghparast‐Bidgoli et al. 2013). In cases of oral and maxillofacial injuries, the number of fracture lines and the use of intermaxillary fixation were identified as independent risk factors for extended hospitalization among injured cyclists and motorcyclists (Hirobe et al. 2021). Specifically regarding treatment duration—as examined in the present study—the number of injured teeth and the use of intermaxillary fixation have previously been reported as independent predictive factors for treatment periods exceeding 1 month (Machida et al. 2023). These findings align with the present study, which identified maxillofacial fractures, dislocation of two or more teeth, and fractured teeth as predictors of prolonged treatment duration in children. This represents a novel contribution of the study. In this study, age was not selected as a predictive factor. In adults, degenerative changes in bone that increase fracture susceptibility, along with comorbidities, can contribute to longer treatment durations. However, as this study focused on pediatric patients, age did not influence treatment duration. Because oral and maxillofacial injuries can lead to psychosocial dysfunction, prolonged treatment durations may increase anxiety and emotional concerns. On the basis of these results, oral and maxillofacial surgeons should consider promoting psychological care for pediatric patients who are at risk for longer treatment durations. Additionally, preventive measures should be implemented to reduce the occurrence of these predictive factors and shorten treatment times, ultimately improving quality of life and allowing patients to return to normal activities more quickly.

The findings presented in this study may assist maxillofacial surgeons in the initial assessment of pediatric patients by helping them anticipate the likelihood of maxillofacial fractures, tooth fractures, or dislocations. Recently, concerns have been raised about the increased cancer risk in children due to CT examinations (Frush et al. 2003). According to Frush et al. the estimated risk of a child developing a fatal cancer from radiation exposure is approximately 1 in 1,000 CT scans (Frush et al. 2003). While CT imaging is valuable for evaluating injuries in children, the insights gained from this study may help limit unnecessary CT examinations by guiding more selective imaging decisions. Additionally, the results suggest that involving nurses or psychologists in the emergency care of injured children may improve the allocation of healthcare resources, allowing maxillofacial surgeons to focus on surgical and diagnostic priorities while ensuring psychological support is not overlooked.

This study has several strengths and limitations. The first strength is that because this university hospital is the only facility in the region with 24‐h access to maxillofacial specialists, most pediatric oral and maxillofacial trauma cases in the prefecture are treated here. As a result, the findings are based on a substantial and representative sample, enhancing their reliability. Second, this study is the first to clarify the prevalence of tooth dislocation and fracture across distinct developmental stages in children, revealing age‐related differences that provide new insights for maxillofacial practitioners. Third, we identified independent predictive factors for prolonged treatment duration specifically in pediatric patients—an area that has not been well studied—thereby offering novel guidance for clinicians treating child trauma cases. The first limitation is that the study did not account for comorbid conditions. Some children may have developmental disorders, such as attention‐deficit/hyperactivity disorder, which are associated with riskier behaviors. These cases may need to be analyzed separately in future research. Second, we did not examine individual predispositions to maxillofacial trauma. Factors such as anterior open bite, incomplete lip coverage, and increased overjet are known to increase the risk of dental injury, particularly to the maxillary central incisors (Berti et al. 2015). Future studies should assess these anatomical risk factors in each patient. Third, this study did not include severely injured patients because our hospital is not a designated advanced emergency and critical care center. Patients with severe polytrauma or critical head injuries were therefore excluded. Future research should compare our findings with data from facilities treating more severely injured pediatric populations.

## 5 Conclusion

Most patients (86.0%) sustained oral and maxillofacial trauma due to falls. Soft tissue injuries (63.6%) and tooth dislocations (58.1%) were more common than tooth fractures (17.8%) among children under 15 years of age. Incisors were the most frequently injured teeth. The prevalence of tooth fractures increased with age and was rarely observed in children aged 2 years or younger, while the prevalence of tooth dislocations did not differ significantly between age groups. Maxillofacial fractures, tooth fractures, and the presence of two or more dislocated teeth were identified as independent predictive factors for prolonged treatment duration, with odds ratios and 95% confidence intervals of 14.666 (1.625–132.359), 2.411 (1.099–5.287), and 2.350 (1.208–4.570), respectively. These findings may help dental practitioners improve diagnostic accuracy and treatment planning for pediatric trauma cases, ultimately reducing the long‐term impact of these injuries. Additionally, efforts to increase public awareness and implement preventive measures should be promoted to reduce the incidence of oral and maxillofacial trauma in children.

## Author Contributions

Yuki Sakamoto contributed to the study conception and design, data acquisition, analysis, and interpretation, and drafted the manuscript. Mineko Baba contributed to the data analysis and interpretation, and drafted the manuscript. Shinya Kosinuma, Kazuki Takaoka, and Mami Nakamura contributed to the data acquisition and interpretation, and drafted the manuscript. Masahito Hitosugi contributed to the study conception and design, data acquisition, analysis, and interpretation, and drafted the manuscript.

## Ethics Statement

This study followed the Declaration of Helsinki on medical protocol and ethics and was approved by the Ethics Committee of Shiga University of Medical Science (R2022‐101).

## Consent

Because this was a retrospective study, open consent was obtained through an opt‐out process.

## Conflicts of Interest

The authors declare no conflicts of interest.

## Data Availability

The data that support the findings of this study are available from the corresponding author upon reasonable request.
